# The Potential of Twendee X^®^ as a Safe Antioxidant Treatment for Systemic Sclerosis

**DOI:** 10.3390/ijms25053064

**Published:** 2024-03-06

**Authors:** Fukka You, Carole Nicco, Yoshiaki Harakawa, Toshikazu Yoshikawa, Haruhiko Inufusa

**Affiliations:** 1 Division of Anti-Oxidant Research, Life Science Research Center, Gifu University, Yanagito 1-1, Gifu 501-1194, Japan; y@antioxidantres.jp (F.Y.); harakawa@antioxidantres.jp (Y.H.); 2 Anti-Oxidant Research Laboratory, Louis Pasteur Center for Medical Research, Tanakamonzen-cho 103-5, Sa-kyo-ku, Kyoto 606-8225, Japan; 3 Université Paris Cité, 45 Rue des Saints-Pères, 75006 Paris, France; 4 Louis Pasteur Center for Medical Research, Tanakamonzen-cho 103-5, Sakyo-ku, Kyoto 606-8225, Japan; toshi@yoshikawalab.jp; 5 School of Medicine, Kyoto Prefectural University of Medicine, Kajii-cho, Kawaramachi-Hirokoji, Kamigyo-ku, Kyoto 602-8566, Japan

**Keywords:** oxidative stress, Twendee X^®^, antioxidant, systemic sclerosis, autoimmunity

## Abstract

Systemic sclerosis (SSc) is an autoimmune disease characterized by systemic skin hardening, which combines Raynaud’s phenomenon and other vascular disorders, skin and internal organ fibrosis, immune disorders, and a variety of other abnormalities. Symptoms vary widely among individuals, and personalized treatment is sought for each patient. Since there is no fundamental cure for SSc, it is designated as an intractable disease with patients receiving government subsidies for medical expenses in Japan. Oxidative stress (OS) has been reported to play an important role in the cause and symptoms of SSc. HOCl-induced SSc mouse models are known to exhibit skin and visceral fibrosis, vascular damage, and autoimmune-like symptoms observed in human SSc. The antioxidant combination Twendee X^®^ (TwX) is a dietary supplement consisting of vitamins, amino acids, and CoQ10. TwX has been proven to prevent dementia in humans with mild cognitive impairment and significantly improve cognitive impairment in an Alzheimer’s disease mouse model by regulating OS through a strong antioxidant capacity that cannot be achieved with a single antioxidant ingredient. We evaluated the effectiveness of TwX on various symptoms of HOCl-induced SSc mice. TwX-treated HOCl-induced SSc mice showed significantly reduced lung and skin fibrosis compared to untreated HOCl-induced SSc mice. TwX also significantly reduced highly oxidized protein products (AOPP) in serum and suppressed Col-1 gene expression and activation of B cells involved in autoimmunity. These findings suggest that TwX has the potential to be a new antioxidant treatment for SSc without side effects.

## 1. Introduction

Systemic sclerosis (SSc) is an autoimmune disease of unknown cause [1,2,3], a chronic disease characterized by vascular and immune dysfunction, and fibrosis of the skin and internal organs caused by excessive collagen deposition by activated fibroblasts [4,5,6,7]. Pulmonary fibrosis and pulmonary arterial hypertension contribute to high mortality [8]. The pathogenesis of SSc is very complex, involving both innate and adaptive immune responses in its development and progression [2,9]. Autoimmune diseases are chronic, refractory diseases stimulated primarily by immune, hormonal, environmental, and genetic factors [10]. Most autoimmune diseases are chronic diseases that are long-lasting or in some cases follow patients throughout their lives, and specific treatment methods have not yet been established [11]. Although the mechanisms that cause clinical manifestations in SSc are also unknown [12], reactive oxygen species (ROS) are well known to be involved in the etiology of SSc [13,14,15,16,17,18,19,20]. Raynaud’s phenomenon (RP) occurs in 90% of SSc patients. After RP, patients may present with severe ischemia and ulceration, resulting in increased morbidity and mortality and decreased quality of life [21,22]. RP causes frequent episodes of hypoxia–reperfusion [23], resulting in a positive feedback effect of luminal narrowing and ischemia, promoting the generation of ROS and free radicals. ROS can modulate cell activation and proliferation, and fibroblasts and endothelial cells are selectively targeted in SSc. Such an oxidative environment further exacerbates the disease by triggering endothelial damage, intimal thickening, and fibrosis. Ischemia and reperfusion induce oxidative stress (OS) [24,25] and inactivation of antioxidant enzymes [26]. In addition, skin fibroblasts in SSc patients have been shown to elevate ROS [27], which triggers collagen synthesis [15,28], emphasizing the significant role of OS in SSc.

Twendee X^®^ (TwX) is an antioxidant combination comprising eight active ingredients: vitamin C, l-glutamine, niacin, l-cystine, coenzyme Q10, vitamin B2, succinic acid, and fumaric acid [29]. Despite being a dietary supplement, it has undergone and passed stringent safety tests required for pharmaceuticals including chromosomal aberration test, toxicity test, and mutation test. 

TwX has demonstrated various beneficial effects including protecting mitochondria, increasing ATP production, reducing blood OS, maintaining autophagy and neurogenesis, and elongating telomeres [30]. The effect of TwX on cognitive function has also been studied in varying disease models. A multicenter randomized, double-blind, placebo-controlled intervention clinical trial has demonstrated its potential to prevent dementia in Japanese patients with mild cognitive impairment (MCI) [31]. Moreover, TwX has shown positive outcomes in mouse models of Alzheimer’s disease (AD) with chronic cerebral hypoperfusion (CCH + APP23 mice); motor coordination and working memory were improved and hippocampal neurons were restored. Other outcomes derived from TwX include significant improvement of cognitive impairment, reduction of Aβ pathology and neuronal loss, and alleviation of neural inflammation and OS [32,33]. 

In a mouse model of ischemic stroke, pretreatment with TwX (20 mg/kg/d) for 14 days not only reduced infarct size but also decreased the expression of OS markers and tumor necrosis factor-α (TNF-α) and inflammation markers [34]. Recent reports have also suggested the potential of TwX to enhance the quality of life in dementia patients by influencing gut microbiota [35]. The effects of TwX shown in previous studies could be effective enough to control OS in SSc. The aim of this study was to investigate the effects of TwX on various human-like SSc symptoms [36,37] in mice that were injected with HOCl, an oxidation accelerator, into their dermis [36,38,39,40,41,42].

## 2. Results and Discussion

### 2.1. Reduction of OS in HOCl-Induced SSc by TwX

**Figure 1:**
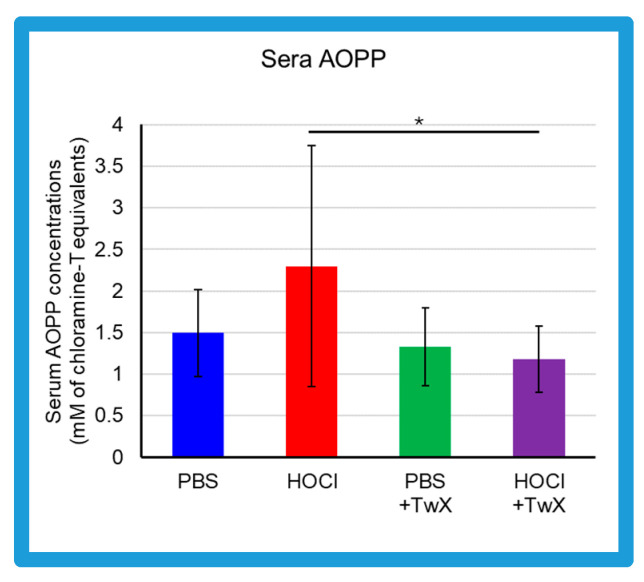
Effect of Twendee X^®^ on sera redox status. Concentrations of advanced oxidation protein products (AOPP) in the sera from mice (mM of chloramine T equivalent). Each box represents mean ± SD from n = 10 individual mice. Kruskal–Wallis test with Dunn’s multiple comparison test was used for statistical analysis). * *p* < 0.05.

Advanced oxidation protein products (AOPP) in the sera of HOCl-induced SSc mice were elevated compared to the control group treated with PBS (PBS control) (+54% (Not significant)) (Figure 1). On the other hand, in serum of PBS control group and HOCl-induced SSc mice treated with TwX (20 mg/kg/d) (PBS + TwX, HOCl + TwX, respectively), AOPP levels were lower than those of the PBS control group and HOCl-induced SSc mice, respectively (PBS vs. PBS + TwX: −11% (Not significant), HOCl vs. HOCl + TwX: −49% (*p* < 0.05)) (Figure 1).

Serum AOPP concentrations in SSc patients are usually reported to be higher than in healthy subjects [13,43,44]. This was also observed in the present study; HOCl-induced SSc mice had higher AOPP than PBS-treated mice. AOPP is a marker of OS. Serum AOPP is involved in the production of ROS and regulates fibroblast proliferation in SSc patients [13]. HOCl-induced AOPP (HOCl-AOPP) induces H_2_O_2_ rather than NO and activates both endothelial cells and fibroblasts, and a dose-dependent proliferative response can be observed [13]. In fact, serum from SSc patients with RP shows significantly higher levels of H_2_O_2_ and proliferation of NIH3T3 fibroblasts compared to serum from SSc patients without RP and healthy subjects [13]. Additionally, decreased concentrations of antioxidants such as ascorbic acid, α-tocopherol, and β-carotene, as well as lower selenium levels have been reported in SSc patients [26]. This increase in ROS and deficiency in antioxidant capacity increases OS and contributes to the development of free-radical-mediated damage. This elevated OS is further exacerbated not only by inflammatory processes but also by frequent reperfusion injury such as that seen in RP [45]. Vascular changes including microvascular endothelial injury, vasospasm tendency with an inadequate vasodilatory response, changes in the coagulation/fibrinolytic system, and proliferation of intimal cells leading to microvascular system occlusion are significant events in the early stages of SSc, and it highlights the crucial role of OS in the early stages of SSc [46] and in the progression and worsening of the disease.

Based on the above, it is quite possible that reducing AOPP may suppress H_2_O_2_, and reducing OS at some point is one pathway to alleviate the various symptoms of SSc. TwX has been shown to protect cells and mitochondria by lowering cellular and mitochondrial ROS levels and increasing Mn-SOD and Cu/Zn-SOD activity [30]. These effects significantly suppress OS caused by blood hydro-peroxides and other substances in CCH + APP23 mice and in OPP rats treated with orthophenyl phenol (OPP), a fruit and another preservative that induces ROS in the body [32,33,35]. In the present study, TwX affected OS to significantly reduce HOCl-derived oxidative damage.

### 2.2. Inhibition of HOCl-Induced Skin and Lung Fibrosis by TwX

**Figure 2:**
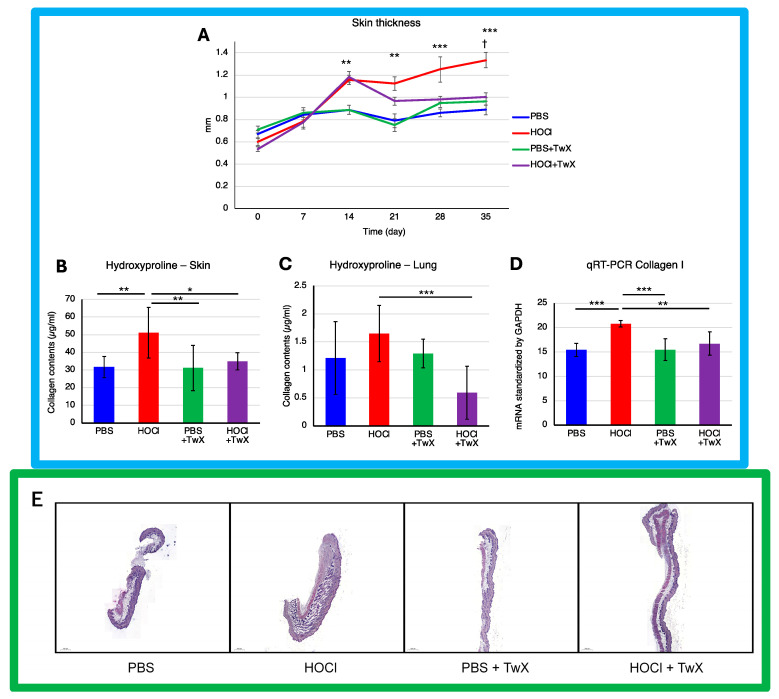
Effect of Twendee X^®^ on fibrosis parameters. (**A**) Change in skin fold thickness in millimeters from day 1 to day 36, measured weekly (n = 10). **: *p* < 0.01, ***: *p* < 0.001 (PBS vs. HOCl), †: *p* < 0.05 (HOCl vs. HOCl + TwX). (**B**) Collagen type I levels in skin (mg/punch biopsy) and (**C**) in lung (mg/lobe biopsy) were evaluated by hydroxyproline dosage. Each box represents mean ± SD from n = 10 individual mice. * *p* < 0.05; ** *p* < 0.01; *** *p* < 0.001. (**D**) Relative mRNA level of Collagen-1. Results were standardized by GAPDH. Each box represents mean ± SD from n = 10 individual mice. ** *p* < 0.01; *** *p* < 0.001. (**E**) Representative H&E dyed skin sections of 6 μm, showing enhanced fibrosis in mice. Photographs were taken with a Nikon Eclipse 80i microscope (Nikon Instruments, Inc., Melville, NY, USA). Original magnification ×20. The scale represents 500 μm. All data are expressed as the mean ± SD. Kruskal–Wallis test with Dunn’s multiple comparison test was used for all statistical analysis.

Continuous intradermal injection of HOCl solution into mice replicates human SSc, inducing local and systemic fibrosis, inflammation, autoimmunity, and vascular injury [39]. The skin thickness (Figure 2A) and hydroxyproline (OH proline) levels in the skin (Figure 2B) of HOCl-induced SSc mice were significantly higher than those in the control group treated with PBS (+50% (*p* < 0.001) for dermal thickness on day 35 and +61% (*p* < 0.01) for OH proline concentration). This indicated elevated collagen levels and increased Col1 gene expression (+34% (*p* < 0.001)) (Figure 2D). However, treating these mice with TwX (20 mg/kg/d) significantly suppressed OH proline levels (−32% (*p* <0.05) for the skin, −64% (*p* < 0.001) for the lung) and Col1 mRNA expression (−20% (*p* < 0.01)), thereby suppressing collagen accumulation (Figure 2B–D). Further observations, confirmed by histopathological images, showed that skin thickness induced by HOCl was gradually suppressed from day 14 onward and significantly reduced to a thickness similar to that of PBS + TwX mice from day 20 onward (−25% on day 35 (*p* < 0.05)) (Figure 2A,E). Similarly, the lungs of HOCl-induced SSc mice showed high concentrations of OH proline (+36% (Not significant)) (Figure 2C), and TwX significantly reduced this collagen accumulation to lower levels than in PBS control mice (−64%, *p* < 0.001).

Several human skin pathologies, including SSc, are associated with significant redox imbalance at the cellular level [13,47,48,49]. Antioxidant administration has also been reported to suppress skin and pulmonary fibrosis in SSc [50,51,52]. One example is Edaravone, a novel, free radical scavenger and neuroprotective agent used in the treatment of acute embolic stroke in humans [53]. The skin fibrosis score in bleomycin-induced mouse models (BLM mice) increases over time, and while Edaravone significantly reduced dermal skin thickness, it did not lower to the level of the control mice [50]. TwX also had an effect on skin and lung fibrosis in HOCl-induced SSc mice. Although the effect of TwX on dermal thickening was not observed in the early stages of HOCl treatment, the effect emerged gradually and eventually decreased dermal thickness to a state close to that of the PBS control mice.

Although TwX also has free radical scavenging [54] and neuroprotective effects [30], it may be necessary to use more immediate agents to improve skin thickening from the initial stage. OS scavenging could lead to a rational targeted therapeutic approach for SSc; however, the complexity and repetitive nature of the cascading system in SSc make it impossible to achieve efficacy with a treatment derived from a single antioxidant [26]. The composition of TwX, which includes eight high-efficacy antioxidants, may underlie its effectiveness in alleviating SSc symptoms to a state similar to the PBS control mice. The same could be true for collagen content in skin and lungs.

**Figure 3:**
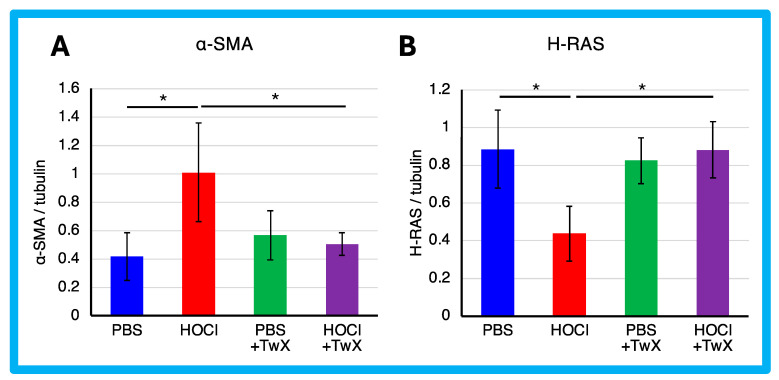
Effect of Twendee X^®^ on fibroblast differentiation. (**A**) α-SMA and (**B**) H-Ras in skin were evaluated by Western blot. Results were normalized to tubulin. Each box represents mean ± SD from n = 10 individual mice. Kruskal–Wallis test with Dunn’s multiple comparison test was used for all statistical analysis. * *p* < 0.05.

The expression of α-SMA protein was significantly enhanced in HOCl-induced SSc mice (+142% (*p* < 0.05)) (Figure 3A). However, TwX significantly reduced it to a level similar to that of PBS control mice (−50% (*p* < 0.05)) (Figure 3A). 

α-SMA is expressed primarily in vascular smooth muscle and is involved in the differentiation of fibroblasts into myofibroblasts, which are responsible for the production of extracellular matrix in fibrotic diseases such as SSc. It has been reported that the development of ROS in SSc fibroblasts increases the expression of type 1 collagen and α-SMA genes [55], while the expression of type I collagen and α-SMA genes also activates ROS [15,28]. In other words, a vicious cycle between the pathology and ROS is indicated. TwX suppressed fibrosis in SSc by decreasing α-SMA as well as ROS. This suggests that TwX is promising as an antioxidant compound to inhibit fibrosis by breaking the vicious cycle between pathology and ROS.

In contrast, the expression of H-Ras protein was significantly reduced in HOCl-induced SSc mice (−51% (*p* < 0.05)) (Figure 3B). H-Ras proteins are primarily involved in the regulation of cell division. When the protein binds to GDP, it does not relay signals to the cell’s nucleus to divide. H-Ras-GTPase proteins are activated for example during renal fibrosis and play crucial roles in regulating both cell proliferation and TGF-β-induced epithelial–mesenchymal transition [56]. The results suggest that normal homeostatic functions may act to protect cells from HOCl-induced excessive division, resulting in a decrease in H-Ras. On the other hand, this decrease was not observed in PBS control or HOCl + TwX mice, likely due to the absence of induced fibrosis. We speculate that TwX prevented fibrosis before the normal homeostatic function was activated in H-Ras.

### 2.3. Effects on Inflammation and Immunity in SSc

**Figure 4:**
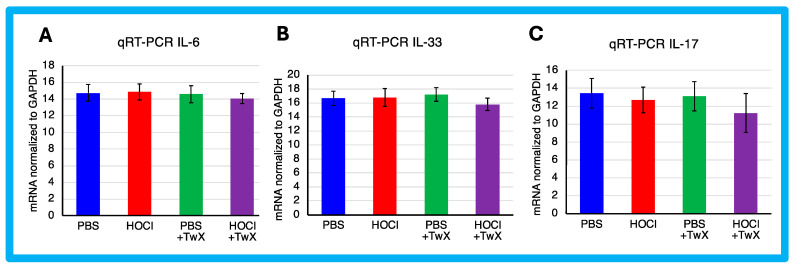
Effect of Twendee X^®^ on Collagen and cytokines expression. Relative mRNA level of (**A**) Il-6, (**B**) Il-33, and (**C**) Il-17 mRNA levels in skin evaluated by qRT-PCR. Results were normalized to GAPDH. Each box represents mean ± SD from n = 10 individual mice. Kruskal–Wallis test with Dunn’s multiple comparison test was used for all statistical analysis. No significant difference was observed.

HOCl-induced SSc mice have been reported to exhibit systemic immune cell infiltration and release of inflammatory mediators [57]. In this study, HOCl-induced SSc mice showed a trend towards higher expression of Il-6 and Il-33 mRNA, but the change was not significant compared to PBS-treated mice (IL-6: +1%, IL-33: +1%). Il-17 showed a decreasing trend compared to the PBS-treated mice (−6%), although the difference was not significant. However, TwX treatment decreased Il-6, Il-33, and Il-17 mRNA expression in SSc mice compared to HOCl-induced SSc mice (IL-6: −5% (Not significant), IL-33: −6% (Not significant), IL-17: −12% (Not significant)) (Figure 4A–C).

In the present study, there was no significant increase in the expression of inflammatory cytokines in HOCl-induced SSc mice. However, since TwX has been shown to suppress inflammation in CCH + APP23 mice [32,33], it may have caused the same result in SSc.

**Figure 5:**
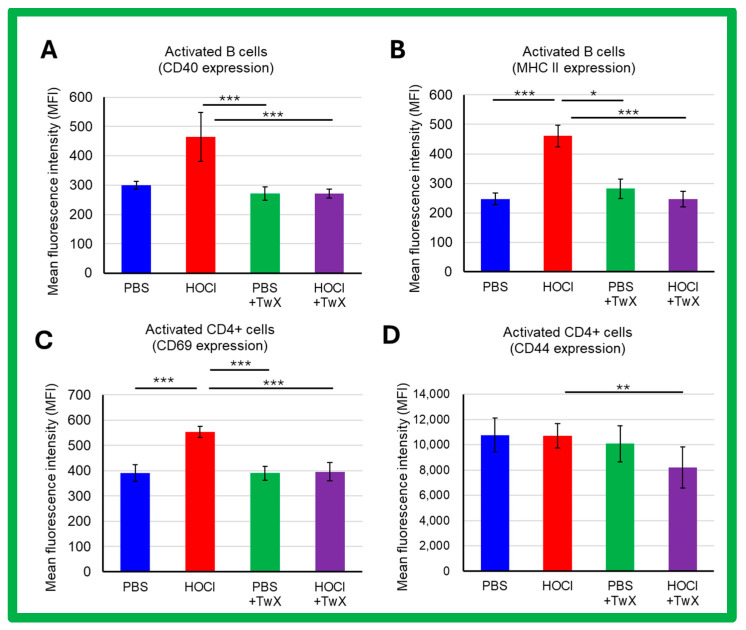
Effects of Twendee X^®^ on B and T CD4+ cells activation assessed by flow cytometry in SSc mice. The side-scatter (SSC) and the forward-scatter channels (FSC) were used to gate the leukocytes. A total of 100,000 events were accumulated for each sample. Doublets were excluded with FSC-A and FSC-H channels. (**A**,**B**) Activation of B220+ cells assessed by CD40 expression (**A**) and MHC II expression (**B**). (**C**,**D**) Activation of CD4+ T cells assessed by CD69 (**C**) and CD44 (**D**) expression. Each box represents mean ± SD from n = 10 individual mice. Kruskal–Wallis test with Dunn’s multiple comparison test was used for all statistical analysis. * *p* < 0.05; ** *p* < 0.01; *** *p* < 0.001.

SSc is a systemic autoimmune disease with chronic activation of adaptive immunity by nuclear autoantigens. In HOCl-induced SSc mice, activation of splenic B cells and CD4+ T cells (CD69 expression) was increased compared to PBS control mice (CD40: +55% (Not significant), MHCII: +87% (*p* < 0.001), CD69: +42% (*p* < 0.001), CD44: −1% (Not significant)). TwX significantly reduced these activations (CD40: −42% (*p* < 0.001), MHCII: −47% (*p* < 0.001), CD69: −29% (*p* < 0.001), CD44: −23% (*p* < 0.01)) (Figure 5A–D).

**Figure 6:**
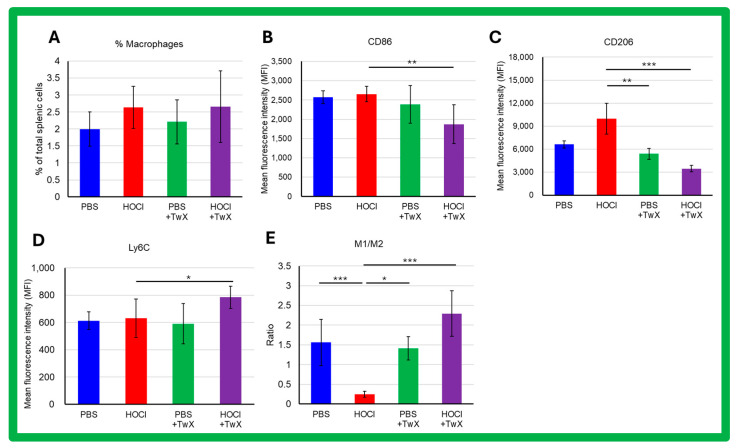
Flow cytometric analysis of the effects of Twendee X^®^ on splenic macrophage phenotype in SSc mice. Splenic macrophages were gated on CD11b- and F4/80-positive cells among CD45-positive. A total of 100,000 events were accumulated for each sample. Doublets were excluded with FSC-A and FSC-H channels. (**A**) Percentage of splenic macrophages among total splenic cells. Data represent the percentage and SD. (**B**–**D**) Flow cytometric analysis of CD86, Ly6C, and CD206 expression on splenic macrophages. (**E**) Ratio of M1/M2 macrophages’ frequency. M1 macrophages were defined as B220-F4/80+CD11b+Ly6CHighCD206- and M2 macrophages as B220-F4/80+CD11b+Ly6cLowCD206+. Each box represents mean ± SD from n = 10 individual mice. Kruskal–Wallis test with Dunn’s multiple comparison test was used for all statistical analysis. * *p* < 0.05; ** *p* < 0.01; *** *p* < 0.001.

Activated macrophages play a key role in triggering and perpetuating the inflammatory process and in developing fibrosis during SSc. The frequency of splenic macrophages was increased by induction of HOCl (+32% (Not significant)) (Figure 6A). The expression of CD86 and Ly6C showed an increasing trend in macrophages of HOCl-induced SSc mice compared to controls (CD86: +3%, Ly6C: +3%, Not significant) (Figure 6B,D); in the expression of CD206, there was an increase compared to controls (+51% (Not significant)) (Figure 6C). Interestingly, HOCl + TwX mice showed a significant decrease in CD86 and CD206 expression in their splenic macrophages (CD86: −29% (*p* < 0.01), CD206: −65% (*p* < 0.001)) (Figure 6B,C), while Ly6C expression was notably increased (+24% (*p* < 0.05), Figure 6D). In addition, we observed a polarization toward the M2 profibrotic macrophage phenotype (*p* < 0.001, Figure 6E) in HOCl-induced SSc mice that was reversed in the HOCl + TwX mice (*p* < 0.001).

Macrophages are inflammatory cells that produce other immune mediators and cytokines with both protective and pro-inflammatory functions [58]. In SSc, macrophages also play an important role in the disease pathogenesis. When macrophages are activated, they release mediators and express surface markers, and normally the activation of both alternative macrophages (M2) and inflammatory macrophages (M1) is associated with SSc [59]. Macrophages are the predominant immune cell population in SSc pathology, and their dysfunction leads to abnormal repair and regeneration with runaway inflammatory mediators and growth factors [60]. Excessive accumulation of M2 macrophages is closely associated with fibrosis [61,62], which results from the abnormal accumulation of extracellular matrix (ECM) components such as collagen and fibronectin. ECM promotes wound healing and tissue repair in mild tissue injury; however, in severe injury, excessive accumulation of ECM can disrupt tissue structure and lead to organ dysfunction [63]. Thus, macrophages play a crucial role in fibrosis pathogenesis [64,65], especially M2a macrophages, which significantly promote fibrosis progression [62,66]. The present study suggests that a shift in macrophage balance toward the M2 phenotype occurred during the chronic phase (after the 21st day of the experiment) in HOCl-induced SSc mice. However, the trend was reversed when TwX was administered, resulting in the accumulation of M1 macrophages over M2 macrophages, thereby slowing the fibrotic process.

## 3. Materials and Methods

### 3.1. Materials

TwX is a compound consisting of the following active ingredients: l-glutamine (34.6 wt% Fujifilm Wako Pure Chemicals Co., Ltd., Osaka, Japan), ascorbic acid (34.2 wt% Fujifilm Wako Pure Chemicals Co., Ltd., Osaka, Japan), l-cystine (18.2 wt% Fujifilm Wako Pure Chemicals Co., Ltd., Osaka, Japan), coenzyme Q10 (3.6 wt%; AQUA Q10 P40-NF, Nissin Pharmaceutical, Tokyo, Japan), succinic acid (3.6 wt%, Sigma-Aldrich Co., St. Louis, MO, USA), fumaric acid (3.6 wt%, Sigma-Aldrich Co., St. Louis, MO, USA), riboflavin (1.5 wt%; Bislase inj; Toa Eiyo, Tokyo, Japan), and niacin amid (0.7 wt%, Sigma-Aldrich Co., St. Louis, MO, USA). TwX mixture was dissolved in sterile water and stored at 4 °C until use.

### 3.2. Chemically Inducing SSc in Mice In Vivo

The animal protocol for this study was reviewed and approved by the local ethic committee (Comité d’Ethique en matière d’Expérimentation animale Paris Descartes CEEA34, Animal facilities C75-14-05, Agreement DAP 2019080816497350 #26065). Six-week-old female BALB/c mice were purchased from Janvier Labs (Le Genest-Saoint-Isle, France). Mice were randomly distributed into experimental and PBS control groups (n = 10). The experimental groups included the HOCl-induced SSc mice group (HOCl, n = 10), the group given PBS and TwX (PBS + TwX, n = 10), and the TwX-treated HOCl-induced SSc group (HOCl + TwX, n = 10).

Subcutaneous injections of 200 µL of HOCl into the back of the mice were administered once daily for 6 weeks, as previously described [38,39]. The PBS control group and PBS + TwX group received injections of 100 μL of sterilized PBS.

One month before and during the 6 weeks of HOCl injections, the mice consumed plain tap water or water with TwX ad libitum (20 mg/kg/d). Two days after the final injection, the animals were euthanized by cervical dislocation, and samples of sera, spleen, lungs, and skin biopsies were collected. Tissue samples were fixed in 10% acetic acid formol for histopathological analysis.

### 3.3. Evaluating Fibrosis

Fibrosis of the skin was assessed weekly in vivo, by measuring the dermal thickness of the shaved backs of the mice. This assessment was conducted under double-blinded conditions using a caliper and expressed in millimeters once a week until the end of the experiment.

### 3.4. Serum AOPP Measurement

Serum AOPP concentration was measured by spectrophotometry as previously described [44]. Assays of AOPP in sera were diluted (1:5) in PBS and distributed (200 μL) onto a 96-well plate with 10 μL of 1.16 M potassium iodide. Calibration used a twofold dilution series of chloramine-T solution within a range of 0 to 100 mM. The absorbance was read at 340 nm on a microplate reader (Fusion; PerkinElmer, Wellesley, MA, USA), and AOPP concentration was expressed as mM of chloramine-T equivalents.

### 3.5. Collagen Content Measurement

The collagen content was assessed in the skin and lungs using the OH-proline content evaluation as recommended by Woessner [67]. Briefly, after completing punch biopsies (5 mm diameter), the samples were incubated in HCl (6 M) for 3 h at 120 °C. The pH of the samples was adjusted to 7 and then mixed with chloramine T (0.06 M) and incubated for 20 min at room temperature. Perchloric acid (3.15 M) and p-dimethylaminobenzaldehyde (20%) were then added, and samples were incubated for an additional 20 min at 60 °C. The absorbance was determined at 557 nm with a Fusion microplate spectrophotometer (PerkinElmer, Wellesley, MA, USA).

### 3.6. Histopathologic Analysis

A 5 μm thick tissue section was prepared from the mid-portion of paraffin-embedded lung and skin sample and stained with hematoxylin and eosin. Slides were examined by standard brightfield microscopy (Olympus BX60, Tokyo, Japan) by a pathologist who was blinded to the animal’s group assignment. 

### 3.7. Evaluation of Fibroblasts

α-SMA and H-Ras proteins expressions in mouse skin were analyzed. Skin samples were mixed in RIPA lysis buffer. Proteins (30 μg per well) were subjected to 10% polyacrylamide gel electrophoresis, transferred onto nitrocellulose membranes, blocked with 5% nonfat dry milk in TBS-T, then incubated overnight at 4 °C with an anti-mouse α-SMA (Abcam, Cambridge, UK. ref ab5694, dilution 1:500) or an anti-H-Ras antibody (Santa Cruz Biotechnology, Dallas, TX, USA. ref sc-53959, dilution 1:500). The membranes were then washed and incubated for 1 h with an HRP-conjugated antibody (Invitrogen, Carlsbad, CA, USA) (goat anti-rabbit ref A27036 (dilution 1:5000), goat anti-mouse ref A28177 (dilution 1:10,000)). Tubulin and B actin were used to standardize the OD values obtained for the different samples.

### 3.8. Evaluation of mRNA Expressions

The qRT-PCR technique was conducted as follows: the skin samples taken from the four groups of mice were immediately frozen in liquid nitrogen. Total RNA was extracted from the mouse tissue using the Trizol reagent (Invitrogen, Carlsbad, CA, USA), according to the manufacturer’s instructions.

The amplified genes were col 1 (collagen 1) 1, Il-33, Il-6, and Il-17. The cDNA was synthesized using the Maxima H Minus cDNA Synthesis Master Mix kit (Thermo Fisher Scientific, Carlsbad, CA, USA). The qPCR was performed using the SensiFAST SYBR No-ROX kit (Bioline, Memphis, TN, USA). The transcription levels of the selected genes were quantified in a LightCycler480^®^ (Roche, San Francisco, CA, USA) thanks to calibration samples (serial dilution) and normalized to two reference genes (GAPDH). The primer sequences used for qPCR are as follows: mm-Col1a1_F GCTCCTCTTAGGGGCCACT, mm-Col1a1_R CCACGTCTCACCATTGGGG, IL-17_F CTG CTG AGC CTG GCG GCT AC, IL-17_R CAT TGC GGT GGA GAG TCC AGG G, IL-33_F ACT ATG AGT CTC CCT GTC CTG, IL-33_R GCT TCA AAG GGG TGA CGT, IL-6_F GAG GAT ACC ACT CCC AAC AGA CC, IL-6_R AAG TGC ATC ATC GTT GTT CAT ACA.

### 3.9. Evaluating Immune Status

Spleen cell suspensions were prepared after hypotonic lysis of erythrocytes in potassium acetate solution and three washes in complete RPMI medium. For each mouse, splenocytes were quantified using a Malassez counting chamber. Cells were then incubated with an antibody at 4 °C for 30 min in the dark in PBS with 2% normal FBS. Flow cytometry was performed using a FACS Fortessa II flow cytometer (BD Biosciences, Franklin Lakes, NJ, USA), according to standard techniques. To characterize splenic cells, the monoclonal antibodies used spleen cell suspensions prepared after hypotonic lysis of erythrocytes in potassium acetate solution and three washes in complete RPMI medium. For each mouse, splenocytes were quantified using a Malassez counting chamber. Cells’ suspensions were incubated for 20 min with the following antibodies. All antibodies were obtained from Biolegend (San Diego, CA, USA) and used at a dilution of 1:100 unless otherwise mentioned: CD3 FITC, CD4 APC Fire 750 (BD Biosciences, San Jose, CA, USA), CD8 BV 605, CD69 PEDazzle594, CD40 PercPCy5.5, B220 APC, CD44 BV650, MHC II eFluor450 (DAPI) (eBioscience, San Diego, CA, USA), F4/80 BV711 (dilution 1:200), CD11b PercP Cy5.5, CD80 BV421, CD86 FITC, CD206 Alexa Fluor 647 (BD Biosciences, San Jose, CA, USA. Dilution 1:200), Ly6C PECy7.

### 3.10. Statistical Analysis

All the results are presented as the means ± SDs. Kruskal–Wallis test with Dunn’s multiple comparison test was used for all statistical analysis. A *p* value less than 0.05 was considered statistically significant.

## 4. Conclusions

The HOCl-induced SSc model mice used in this study were artificially reproduced, and not all of them are applicable to human SSc symptoms. However, among the SSc induction models using different reagents, the HOCl-induced mouse model used in this study is the model that best reproduces human SSc symptoms. The present study suggests that TwX may delay the transition to the chronic phase in the HOCl-induced SSc mouse model by reducing skin thickening and suppressing skin and lung fibrosis. This indicates that TwX can be expected to alleviate symptoms against SSc in humans. Furthermore, previous studies have suggested that TwX is promising as a safe and effective antioxidant treatment for SSc by modulating OS. Future clinical studies in humans are needed to confirm this conclusion.

## Data Availability

The data are available upon proper request.

## References

[1] Ramos-Casals M., Fonollosa-Pla V., Brito-Zerón P., Sisó-Almirall A. (2010). Targeted therapy for systemic sclerosis: How close are we?. Nat. Rev. Rheumatol..

[2] Zuo X., Zhang L., Luo H., Li Y., Zhu H. (2017). Systematic approach to understanding the pathogenesis of systemic sclerosis. Clin. Genet..

[3] Yang H., Cheong S., He Y., Lu F. (2023). Mesenchymal stem cell-based therapy for autoimmune-related fibrotic skin diseases-systemic sclerosis and sclerodermatous graft-versus-host disease. Stem Cell Res. Ther..

[4] LeRoy E.C., Medsger T.A. (2001). Criteria for the classification of early systemic sclerosis. J. Rheumatol..

[5] Varga J., Abraham D.J. (2007). Systemic sclerosis: A prototypic multisystem fibrotic disorder. J. Clin. Investig..

[6] Volkmann E.R., Andréasson K., Smith V. (2023). Systemic sclerosis. Lancet.

[7] Tamby M.C., Chanseaud Y., Guillevin L., Mouthon L. (2003). New insights into the pathogenesis of systemic sclerosis. Autoimmunity Rev..

[8] Bhattacharyya S., Wei J., Varga J. (2011). Understanding fibrosis in systemic sclerosis: Shifting paradigms, emerging opportunities. Nat. Rev. Rheumatol..

[9] Zhou B., Zuo X.X., Li Y.S., Gao S.M., Dai X.D., Zhu H.L., Luo H. (2017). Integration of microRNA and mRNA expression profiles in the skin of systemic sclerosis patients. Sci. Rep..

[10] Bieber K., Hundt J.E., Yu X., Ehlers M., Petersen F., Karsten C.M., Köhl J., Kridin K., Kalies K., Kasprick A. (2023). Autoimmune pre-disease. Autoimmun. Rev..

[11] Committee for the Assessment of NIH Research on Autoimmune Diseases, Board on Population Health and Public Health Practice, Health and Medicine Division, National Academies of Sciences, Engineering, and Medicine (2022). Enhancing NIH Research on Autoimmune Disease.

[12] Gabrielli A., Avvedimento E.V., Krieg T. (2009). Scleroderma. N. Engl. J. Med..

[13] Servettaz A., Guilpain P., Goulvestre C., Chéreau C., Hercend C., Nicco C., Guillevin L., Weill B., Mouthon L., Batteux F. (2007). Radical oxygen species production induced by advanced oxidation protein products predicts clinical evolution and response to treatment in systemic sclerosis. Ann. Rheum. Dis..

[14] Simonini G., Cerinic M.M., Generini S., Zoppi M., Anichini M., Cesaretti C., Pignone A., Falcini F., Lotti T., Cagnoni M. (1999). Oxidative stress in Systemic Sclerosis. Mol. Cell Biochem..

[15] Sambo P., Baroni S.S., Luchetti M., Paroncini P., Dusi S., Orlandini G., Gabrielli A. (2001). Oxidative stress in scleroderma: Maintenance of scleroderma fibroblast phenotype by the constitutive up-regulation of reactive oxygen species generation through the NADPH oxidase complex pathway. Arthritis Rheum..

[16] Ogawa F., Shimizu K., Muroi E., Hara T., Hasegawa M., Takehara K., Sato S. (2006). Serum levels of 8-isoprostane, a marker of oxidative stress, are elevated in patients with systemic sclerosis. Rheumatology.

[17] Bourji K., Meyer A., Chatelus E., Pincemail J., Pigatto E., Defraigne J.O., Singh F., Charlier C., Geny B., Gottenberg J.E. (2015). High reactive oxygen species in fibrotic and nonfibrotic skin of patients with diffuse cutaneous systemic sclerosis. Free Radic. Biol. Med..

[18] Abdulle A.E., Diercks G.F.H., Feelisch M., Mulder D.J., van Goor H. (2018). The Role of Oxidative Stress in the Development of Systemic Sclerosis Related Vasculopathy. Front. Physiol..

[19] Gabrielli A., Svegliati S., Moroncini G., Amico D. (2012). New insights into the role of oxidative stress in scleroderma fibrosis. Open Rheumatol. J..

[20] Gabrielli A., Svegliati S., Moroncini G., Pomponio G., Santillo M., Avvedimento E.V. (2008). Oxidative stress and the pathogenesis of scleroderma: The Murrell’s hypothesis revisited. Semin. Immunopathol..

[21] Almeida C., Almeida I., Vasconcelos C. (2015). Quality of life in systemic sclerosis. Autoimmune Rev..

[22] Abouyahya I., Liem S.I.E., Amoura Z., Fonseca J.E., Chaigne B., Cutolo M., Doria A., Fischer-Betz R., Guimaraes V., Huizinga T.W.J. (2022). Health-related quality of life in patients with mixed connective tissue disease: A comparison with matched systemic sclerosis patients. Clin. Exp. Rheumatol..

[23] Herrick A.L. (2005). Pathogenesis of raynaud’s phenomenon. Rheumatology.

[24] McCord J.M. (1985). Oxygen-derived free radicals in postischemic tissue injury. N. Engl. J. Med..

[25] Sinning C., Westermann D., Clemmensen P. (2017). Oxidative stress in ischemia and reperfusion: Current concepts, novel ideas and future perspectives. Biomark. Med..

[26] Simonini G., Pignone A., Generini S., Falcini F., Cerinic M.M. (2000). Emerging potentials for an antioxidant therapy as a new approach to the treatment of systemic sclerosis. Toxicology.

[27] Sambo P., Jannino L., Candela M., Salvi A., Donini M., Dusi S., Luchetti M.M., Gabrielli A. (1999). Monocytes of patients with systemic sclerosis (scleroderma) spontaneously release in vitro increased amounts of superoxide anion. J. Investig. Dermatol..

[28] Svegliati S., Cancello R., Sambo P., Luchetti M., Paroncini P., Orlandini G., Discepoli G., Paterno R., Santillo M., Cuozzo C. (2005). Platelet-derived growth factor and reactive oxygen species regulate Ras protein levels in primary human fibroblasts via ERK1/2: Amplification of ROS and Ras in systemic sclerosis fibro-blasts. J. Biol. Chem..

[29] Inufusa H. (2015). Composition for Protection against Cytotoxic Effects. TIMA Foundation. Patent.

[30] You F., Harakawa Y., Yoshikawa T., Inufusa H. (2023). Why Does the Antioxidant Complex Twendee X^®^ Prevent Dementia?. Int. J. Mol. Sci..

[31] Tadokoro K., Morihara R., Ohta Y., Hishikawa N., Kawano S., Sasaki R., Matsumoto N., Nomura E., Nakano Y., Takahashi Y. (2019). Clinical Benefits of Antioxidative Supplement Twendee X for Mild Cognitive Impairment: A Multicenter, Randomized, Double-Blind, and Placebo-Controlled Prospective Interventional Study. J. Alzheimers Dis..

[32] Liu X., Yamashita T., Shang J., Shi X., Morihara R., Huang Y., Sato K., Takemoto M., Hishikawa N., Ohta Y. (2019). Clinical and Pathological Benefit of Twendee X in Alzheimer’s Disease Transgenic Mice with Chronic Cerebral Hypoperfusion. J. Stroke Cerebrovasc. Dis..

[33] Liu X., Yamashita T., Shang J., Shi X., Morihara R., Huang Y., Sato K., Takemoto M., Hishikawa N., Ohta Y. (2019). Twendee X Ameliorates Phosphorylated Tau, α-Synuclein and Neurovascular Dysfunction in Alzheimer’s Disease Transgenic Mice with Chronic Cerebral Hypoperfusion. J. Stroke Cerebrovasc. Dis..

[34] Kusaki M., Ohta Y., Inufusa H., Yamashita T., Morihara R., Nakano Y., Liu X., Shang J., Tian F., Fukui Y. (2017). Neuroprotective Effects of a Novel Antioxidant Mixture Twendee X in Mouse Stroke Model. J. Stroke Cerebrovasc. Dis..

[35] You F., Harakawa Y., Yoshikawa T., Inufusa H. (2023). Controlling Gut Microbiota by Twendee X® May Contribute to Dementia Prevention. Int. J. Mol. Sci..

[36] Morozan A., Joy S., Fujii U., Fraser R., Watters K., Martin J.G., Colmegna I. (2023). Superiority of systemic bleomycin to intradermal HOCl for the study of interstitial lung disease. Sci. Rep..

[37] Maria A.T.J., Toupet K., Maumus M., Rozier P., Vozenin M.C., Le Quellec A., Jorgensen C., Noël D., Guilpain P. (2018). Fibrosis Development in HOCl-Induced Systemic Sclerosis: A Multistage Process Hampered by Mesenchymal Stem Cells. Front. Immunol..

[38] Servettaz A., Goulvestre C., Kavian N., Nicco C., Guilpain P., Chéreau C., Vuiblet V., Guillevin L., Mouthon L., Weill B. (2009). Selective oxidation of DNA topoisomerase 1 induces systemic sclerosis in the mouse. J. Immunol..

[39] Batteux F., Kavian N., Servettaz A. (2011). New insights on chemically induced animal models of systemic sclerosis. Curr. Opin. Rheumatol..

[40] Bagnato G.L., Irrera N., Pizzino G., Santoro D., Roberts W.N., Bagnato G., Pallio G., Vaccaro M., Squadrito F., Saitta A. (2018). Dual αvβ3 and αvβ5 blockade attenuates fibrotic and vascular alterations in a murine model of systemic sclerosis. Clin. Sci..

[41] Bagnato G., Bitto A., Pizzino G., Irrera N., Sangari D., Cinquegrani M., Roberts W.N., Matucci Cerinic M., Squadrito F., Altavilla D. (2013). Simvastatin attenuates the development of pulmonary and cutaneous fibrosis in a murine model of systemic sclerosis. Rheumatology.

[42] Bagnato G., Bitto A., Pizzino G., Roberts W.N., Squadrito F., Altavilla D., Bagnato G., Saitta A. (2015). Propylthiouracil modulates aortic vasculopathy in the oxidative stress model of systemic sclerosis. Vasc. Pharmacol..

[43] Allanore Y., Borderie D., Lemaréchal H., Ekindjian O.G., Kahan A. (2004). Acute and sustained effects of dihydropyridine-type calcium channel antagonists on oxidative stress in systemic sclerosis. Am. J. Med..

[44] Witko-Sarsat V., Friedlander M., Capeillère-Blandin C., Nguyen-Khoa T., Nguyen A.T., Zingraff J., Jungers P., Descamps-Latscha B. (1996). Advanced oxidation protein products as a novel marker of oxidative stress in uremia. Kidney Int..

[45] Al-Adwi Y., Atzeni I.M., Doornbos-van der Meer B., Abdulle A.E., van Roon A.M., Stel A., van Goor H., Smit A.J., Westra J., Mulder D.J. (2023). Release of High-Mobility Group Box-1 after a Raynaud’s Attack Leads to Fibroblast Activation and Interferon-γ Induced Protein-10 Production: Role in Systemic Sclerosis Pathogenesis. Antioxidants.

[46] Murrell D.F. (1993). A radical proposal for the pathogenesis of scleroderma. J. Am. Acad. Dermatol..

[47] Amstad P., Peskin A., Shah G., Mirault M.E., Moret R., Zbinden I., Cerutti P. (1991). The balance between Cu,Zn-superoxide dismutase and catalase affects the sensitivity of mouse epidermal cells to oxidative stress. Biochemistry.

[48] Marut W.K., Kavian N., Servettaz A., Nicco C., Ba L.A., Doering M., Chéreau C., Jacob C., Weill B., Batteux F. (2012). The organotelluride catalyst (PHTE)₂NQ prevents HOCl-induced systemic sclerosis in mouse. J. Investig. Dermatol..

[49] Zhu X., Chu H., Jiang S., Liu Q., Liu L., Xue Y., Zheng S., Wan W., Qiu J., Wang J. (2017). Sirt1 ameliorates systemic sclerosis by targeting the mTOR pathway. J. Dermatol. Sci..

[50] Yoshizaki A., Yanaba K., Ogawa A., Iwata Y., Ogawa F., Takenaka M., Shimizu K., Asano Y., Kadono T., Sato S. (2011). The specific free radical scavenger edaravone suppresses fibrosis in the bleomycin- induced and tight skin mouse models of systemic sclerosis. Arthritis Rheum..

[51] Baral H., Sekiguchi A., Uchiyama A., Nisaa Amalia S., Yamazaki S., Inoue Y., Yokoyama Y., Ogino S., Torii R., Hosoi M. (2021). Inhibition of skin fibrosis in systemic sclerosis by botulinum toxin B via the suppression of oxidative stress. J. Dermatol..

[52] Yao Q., Wu Q., Xu X., Xing Y., Liang J., Lin Q., Huang M., Chen Y., Lin B., Chen W. (2020). Resveratrol Ameliorates Systemic Sclerosis via Suppression of Fibrosis and Inflammation Through Activation of SIRT1/mTOR Signaling. Drug Des. Devel Ther..

[53] Edaravone Acute Infarction Study Group (2003). Effect of a novel free radical scavenger, edaravone (MCI-186), on acute brain infarction: Randomized, placebo-controlled, double-blind study at multicenters. Cerebrovasc. Dis..

[54] Feng T., Yamashita T., Tsunoda K., Matsumoto N., Tadokoro K., Sasaki R., Abe K. (2020). In Vitro Free Radical Scavenging Activities of Dietary Supplements by Electron Spin Resonance. Brain Suppl..

[55] Spadoni T., Svegliati Baroni S., Amico D., Albani L., Moroncini G., Avvedimento E.V., Gabrielli A. (2015). A reactive oxygen species-mediated loop maintains increased expression of NADPH oxidases 2 and 4 in skin fibroblasts from patients with systemic sclerosis. Arthritis Rheumatol..

[56] Grande M.T., Fuentes-Calvo I., Arévalo M., Heredia F., Santos E., Martínez-Salgado C., Rodríguez-Puyol D., Nieto M.A., López-Novoa J.M. (2010). Deletion of H-Ras decreases renal fibrosis and myofibroblast activation following ureteral obstruction in mice. Kidney Int..

[57] Meng M., Tan J., Chen W., Du Q., Xie B., Wang N., Zhu H., Wang K. (2019). The Fibrosis and Immunological Features of Hypochlorous Acid Induced Mouse Model of Systemic Sclerosis. Front. Immunol..

[58] Amico D., Spadoni T., Rovinelli M., Serafini M., D’Amico G., Campelli N., Svegliati Baroni S., Gabrielli A. (2015). Intracellular free radical production by peripheral blood T lymphocytes from patients with systemic sclerosis: Role of NADPH oxidase and ERK1/2. Arthritis Res Ther..

[59] Peng Y., Zhou M., Yang H., Qu R., Qiu Y., Hao J., Bi H., Guo D. (2023). Regulatory Mechanism of M1/M2 Macrophage Polarization in the Development of Autoimmune Diseases. Mediat. Inflamm..

[60] Wynn T.A., Vannella K.M. (2016). Macrophages in tissue repair, regeneration, and fibrosis. Immunity.

[61] Chung E.J., Kwon S., Shankavaram U., White A.O., Das S., Citrin D.E. (2022). Natural variation in macrophage polarization and function impact pneumocyte senescence and susceptibility to fibrosis. Aging.

[62] Jeljeli M., Riccio L.G.C., Doridot L., Chêne C., Nicco C., Chouzenoux S., Deletang Q., Allanore Y., Kavian N., Batteux F. (2019). Trained immunity modulates inflammation-induced fibrosis. Nat. Commun..

[63] Henderson N.C., Rieder F., Wynn T.A. (2020). Fibrosis: From mechanisms to medicines. Nature.

[64] Nakagawa M., Karim M.R., Izawa T., Kuwamura M., Yamate J. (2021). Immunophenotypical characterization of M1/M2 macrophages and lymphocytes in cisplatin-induced rat progressive renal fibrosis. Cells.

[65] Chêne C., Rongvaux-Gaïda D., Thomas M., Rieger F., Nicco C., Batteux F. (2023). Optimal combination of arsenic trioxide and copper ions to prevent autoimmunity in a murine HOCl-induced model of systemic sclerosis. Front. Immunol..

[66] Duan J., Liu X., Wang H., Guo S.W. (2018). The M2a macrophage subset may be critically involved in the fibrogenesis of endometriosis in mice. Reprod. Biomed. Online.

[67] Woessner J.F. (1961). The determination of hydroxyproline in tissue and protein samples containing small proportions of this imino acid. Arch. Biochem. Biophys..

